# Unveiling resilience: coelomic fluid bacteria’s impact on plant metabolism and abiotic stress tolerance

**DOI:** 10.1080/15592324.2024.2363126

**Published:** 2024-06-04

**Authors:** Lamia Yakkou, Sofia Houida, Aicha El Baaboua, Serdar Bilen, Maryam Chelkha, Leyla Okyay Kaya, Abderrahim Aasfar, Fuad Ameen, Sartaj Ahmad Bhat, Mohammed Raouane, Souad Amghar, Abdellatif El Harti

**Affiliations:** a Laboratory of Microbial Biotechnologies and Plant Protection (LBVRN), Faculty of Sciences Agadir, University Ibn Zohr, Agadir, Morocco; b Faculty of Applied Sciences- Ait Melloul, University Ibn Zohr, Agadir, Morocco; c Laboratory of Mycobacteria and Tuberculosis, Institut Pasteur of Morocco, Casablanca, Morocco; d Biotechnology and Applied Microbiology Team, Department of Biology, Faculty of Science, Abdelmalek-Essaadi University, Tetouan, Morocco; e Soil Science and Plant Nutrition Department, Faculty of Agriculture, Ataturk University, Erzurum, Turkey; f Department of Entomology, Cornell University, Cornell AgriTech, Geneva, NY, USA; g Plant and Microbial Biotechnology Center, Moroccan Foundation for Advanced Science, Innovation and Research (MAScIR), Mohammed VI Polytechnic University, Ben Guerir, Morocco; h Department of Botany and Microbiology, College of Science, King Saud University, Riyadh, Saudi Arabia; i River Basin Research Center, Gifu University, Gifu, Japan; j Research Team «Lumbricidae, Improving Soil Productivity and Environment (LAPSE)», Center “Water, Natural Resources, Environment and Sustainable Development, Ecole Normale Supérieure (ENS), Mohammed V University, Rabat, Morocco

**Keywords:** Earthworm, coelomic fluid, PGPR, plant tolerance, abiotic stress, lipidome

## Abstract

Earthworms’ coelomic fluid (CF) has been discovered to possess properties that promote plant development. In particular, the earthworm’s coelomic fluid-associated bacteria (CFB) are the primary factor influencing the plants’ response. To investigate this, we used bacteria isolated from the CF and selected based on different plant growth-promoting traits, in a mesocosm ecosystem that includes plants. This experiment aimed to assess their impact on the metabolism of plants growing under abiotic stress environments (alkaline soil and nitrogen (N), phosphate (P), and potassium (K) deficit) and compare the lipid profiles of plants under the various treatments. We used seven different bacterial species isolated from the CF of *Aporrectodea molleri* and as a plant model *Zea mays* L. For the metabolomic analysis method, we used gas chromatography-mass spectrometry lipidomic. After observing the metabolomic profiles, we found that a few molecular pathways are involved in how plants react to bacterial biostimulants. The bacterial isolates belonging to *Pantoea vagans*, *Pseudomonas aeruginosa*, *Bacillus paramycoides*, and *Bacillus thuringiensis* have led to a significant increase in synthesizing several metabolites belonging to various chemical categories. Contrary to predictions, abiotic stress did not cause a drop in the composition and concentration of lipids in plants treated with the CFB, demonstrating the rigidity of the protective mechanisms. The statistical analysis based on the Pearson method revealed a positive significant correlation between plant growth parameters (length of the aerial part, surface of the leaves, and biomass) and some metabolites belonging to fatty acids, carboxylic acids, benzene derivatives, and alkanes. Moreover, the standard metabolic components of all treatments in much higher concentrations during bacterial treatments than the control treatment suggests that the bacteria have stimulated the overexpression of these metabolic components. According to these results, we could assume that plants treated with CFB exhibit an adaptability of abiotic stress defense mechanisms, which may be attributed to the upregulation of genes involved in lipid biosynthesis pathways.

## 1. Introduction

The soil’s abundant earthworm population creates optimal conditions for the proliferation and heightened activity of specific associated microorganisms. This biological activity significantly influences rhizosphere functions as earthworms consume microorganisms from the rhizosphere, thus playing a crucial part in the ecosystem’s ecological dynamics^1^. Earthworms selectively identify and ingest particular microorganisms at sites with high levels of microbial activity, such as plant roots and organic residues.^2–4^ Therefore, Doube et al. (1994) investigated the feasibility of introducing and distributing beneficial bacteria into soil using earthworms. They emphasized using earthworms for root nodulation when combined with beneficial microbes such as *Pseudomonas sp*. and *Metarhizium sp*. According to the study’s findings, this novel technology drastically modifies soil’s physical, chemical, and biological environment and is highly promising.^5^

Earthworms consume several tons of organic waste per hectare per year while digging down, along with a mixture of soil microorganisms.^1,6–8^ However, digging the underground gallery network would be impossible without producing lubricating and viscous cutaneous excretions, which protect the earthworms against dehydration and facilitate movement by reptation and sliding.^9,10^ The coelomic fluid (CF) represents the majority of these excretions.^11^

In fact, the coelomic fluid would be the perfect place for parasites and pathogens to colonize if it was not protected by a robust immune system.^12–14^ Therefore, this liquid was regarded as aseptic. However, the results of our earlier study, which used a culture-dependent methodology, highlighted the presence of microorganisms in CF.^11,15^ This allowed us to assume that the earthworm tolerates these microorganisms within an associational framework thought to be of the mutualism type.

Research has demonstrated that CF contains antifungal and phytohormonal effects, among other activities, that either directly or indirectly stimulate plant growth. *Fusarium oxysporum*, a harmful fungus to the most significant crops, including wheat, maize, and rice, is negatively impacted by CF.^16^ Also, recent research has indicated that CF may help to improve plant growth metrics and seed germination.^17,18^

Plant-host interactions are among the most challenging and complex biochemical scenarios.^19,20^ Metabolomic approaches are currently being used to assess these interactions.^21,22^ The metabolome’s components represent the mechanism that governs the biochemical phenotype of a cell, tissue, or entire organism.^23^ Utilizing metabolomics in plants like maize has primarily been used to analyze the effects of genetic and environmental factors or to identify any potential differences in maize metabolomes under various biotic and abiotic stresses.^24–26^ Furthermore, plants frequently experience notable changes in the regulation, synthesis, degradation, relocation, or restructuring of lipids when exposed to various types of stress.^27^ Therefore, as a follow-up to our earlier research and tests on the bacteria found in earthworm coelomic fluid, the current work aimed to (i) investigate the impact of these bacteria on the metabolism of plants growing in abiotically stressed environments, as well as (ii) compare the lipid profiles of plants under the various treatments.

## 2. Materials and methods

### 2.1. Earthworm, bacteria, plants, and soil

The Earthworm *Aporrectodea molleri* (Accession number: MT878074) has been identified through genetic analysis by Grupo de Ecología Animal, Universidade de Vigo, Torre Cacti. Lab 97 Vigo, Spain.

In this study, we tested seven bacteria isolated by punction from the coelomic fluid of the earthworm *Aporrectodea molleri*. These bacteria were selected based on different plant growth-promoting traits. The accession numbers of the studied strains are: CFB1 *Buttiauxella gaviniae S1/1–984*^*T*^ (MT576621), CFB2 *Pantoea vagans LMG 24,199*^*T*^ (MT576623), CFB3 *Aeromonas hydrophila JCM1027*^*T*^ (MT576615), CFB4 *Pseudomonas aeruginosa NBRC 12,689*^*T*^ (MT576612), CFB5 *Staphylococcus epidermidis NBRC 100,911*^*T*^ (MT576613), CFB6 *Bacillus paramycoides MCCC 1A04098*^*T*^ (MT576619), CFB7 *Bacillus thuringiensis IAM 12,077*^*T*^ (MT576616).

We used Zea mays L. (DKC7240 silage corn) seed varieties determined as high-yield cultivars in Erzurum ecological conditions, as test crops for this research (https://www.dekalb.com.tr/urun-katalogu/misir-tohumlari/dkc7240). Seeds belonging to these corn varieties were obtained from Dekalb Company, Adana (Turkey). We used the alkaline soil pH = 8 collected from Ataturk University, Research Farm Erzurum, Turkey (Turkey, 39°54‘80“N 41°15’06“E) as a substrate.

### 2.2. Plant mesocosm and inoculum

We sterilized the seed using a 5% H_2_O_2_ solution for 20 minutes. Seeds were then washed with pure water 5 to 6 times. Then, we planted three of these seeds in pots containing autoclaved soil. Each treatment consisting of 3 seeds is conducted in triplicate.^28^ The seeds of control plants (without bacterial inoculation) were soaked in 30 milliliters of a sterile 0.01 M MgSO_4_ solution for 10 minutes, while the treated plants were immersed in 30 ml of a bacterial suspension at 1 × 10^7^ CFU for 10 minutes. We moistened the soil for the control plants with a sterile solution of 0.01 M MgSO_4_, or 1 liter per 3 kg of potting soil.

The cells of the bacterial strains used for inoculation were obtained following culture in Nutrient Broth medium and incubated at 30°C for 48 hours, followed by centrifugation to recover the pellet with a sterile 0.01 M MgSO4 solution. The bacterial solutions were adjusted by diluting up to the same absorbance value with 0.01 M MgSO4, using the turbidimeter (adjusted to 0.6 NTU (nephelometric turbidity unit)), to attain uniform quantities of bacterial cultures for use as inoculation material. The number of bacteria in the whole medium was equal to 1 × 10^7^ CFU ml^−1^ .^29^

The soil was moistened for treated plants with a bacterial suspension at 3 × 10^7^ cells ml^−1^ with a final bacterial concentration of 1 × 10^7^ cells g^−1^ of soil. The seedlings were grown in a greenhouse under a day/night cycle of 15/9 h natural light (ranging from 900 to 1,200 *μ*mol m^−2^ s^−1^), 16–25°C and 55% relative humidity. During the 60-day incubation period, we checked the moisture level of the soils every 2–3 days, to keep the soil moisture content at field capacity. We watered the pots to 55–60% of their soil’s water-holding capacity (SWHC). Fifteen days after sowing, a bacterial booster was carried out on the plants treated with earthworm isolates, and we irrigated each pot with 15 milliliters of the corresponding bacterial suspension. The soil was irrigated with Nitrogen, phosphorus, and potassium-free Hoagland’s solution.^30,31^ The phosphate and potassium sources were replaced by the insoluble forms tricalcium phosphate and Feldspar powder, respectively.

### 2.3. Plant harvest

Plants were harvested 60 days after seeding. We gently removed the plants from soil with finger taps and running water. We separated the shoots and roots and dried using liquid nitrogen.

### 2.4. Metabolite extraction

The extraction technique of Mzibra et al. (2021) was applied. After growing for 60 days, 300 mg of the leaf was homogenized in liquid nitrogen with a mortar and pestle before being put into a glass vial with a stopper. The addition and thorough mixing of 2 mL of precooled to −20°C chloroform solution with dodecane as the internal standard. The mixture was next heated to 85°C for 2 hours on a heating block (Labet International, Edison, USA) and sonicated for 1 hour at 60°C in an ultrasonic sonicator (Branson ultrasonic Sonifier 450, Danbury, USA). After that, 2 mL of methanol was added, mixed, and put back into an ultrasonic sonicator at 60°C for 2 hours. We filtered the extracts through a separating funnel, collected them, and dried them with nitrogen flux.

### 2.5. GC-MS metabolomics analysis

We added 500 L of 6% methanolic HCl (v/v) to the dried residue. The mixture was heated for 30 minutes at 85°C, followed by 15 minutes of vortexing and sonication at 60°C (We repeated the heating and sonication process four times). The mixture was dried using nitrogen flux, and the dried residue was then mixed with 750 mL of chloroform (CHCl_3_) and 250 mL of distilled water in the vortex for one minute. Using a separating funnel, the lower organic phase was transferred to clean vials and kept at −20 for GC-MS analysis.

We performed the metabolomic analysis by gas chromatography (GC) (Agilent 7890A Series GC, USA) coupled to mass spectrometry (MS) equipped with a multimode injector and DB-5 MS column of dimension 30 m × 320 μm × 0.1 μm and ionization by electron impact. We solubilize a volume of 10 μL of sample in 300 μL of chloroform. The column was then filled with 4 μL using helium as the carrier gas at a rate of 4 mL per minute in split mode 1/5. The quadruple and ion source temperatures were 150°C and 230°C, respectively. Starting at 30°C and ending at 360°C, the furnace temperature program was run.

We ascertained the metabolite composition by comparing the peak areas to the dodecane area with a known quantity used as the internal standard. A gain factor of five was used for detection in full scan mode between 30 and 1050 m/z, and the NIST 2017 MS library was used for identification. Sigma-Aldrich provided all of the chemicals used in the study (Spruce, St. Louis, Missouri, USA).

### 2.6. Statistical analysis and bioinformatics

We calculated the concentrations of metabolites extracted in each sample by converting the peak surface area to the concentration of the internal standard. Then, scaling normalization of metabolite data was performed to remove unwanted analytical variation, correct inter- and intra-batch variability, and reduce the influence of outliers.

After normalization, the metabolites identified for each treatment were analyzed by multivariate tests, including heatmap analysis on OriginLab Pro software.

We performed the lipid profiles analysis using the Kyoto Encyclopedia – Genes and Genomes database (KEGG; https://www.genome.jp/kegg/.) and the online analysis software MetaboAnalyst (www.metaboanalyst.ca/).

The variable importance on projection (VIP) score value was obtained from the OPLS-DA model and used to screen the differential metabolites. A high score indicates a strong discriminatory ability.

## 3. Results

We evaluated *in vivo* seven bacteria with a very high PGP (plant growth promotion) capability that were isolated from the coelomic fluid of the earthworm *Aporrectodea molleri*: CFB1 *Buttiauxella gaviniae S1/1–984*^*T*^ (MT576621), CFB2 *Pantoea vagans LMG 24,199*^*T*^ (MT576623), CFB3 *Aeromonas hydrophila JCM1027*^*T*^ (MT576615), CFB4 *Pseudomonas aeruginosa NBRC 12,689*^*T*^ (MT576612), CFB5 *Staphylococcus epidermidis NBRC 100,911*^*T*^ (MT576613), CFB6 *Bacillus paramycoides MCCC 1A04098*^*T*^ (MT576619), CFB7 *Bacillus thuringiensis IAM 12,077*^*T*^ (MT576616).

We carried out a study of metabolomic profiles using gas chromatography and a mass spectrometer (GC-MS) on the leaves and roots of plants treated and untreated with bacteria isolated from coelomic fluid to identify potential metabolites that could explain the impact of various treatments on maize under stress conditions (CFB). Plants inoculated with bacteria (CFB1,…, CFB7) obtained from the coelomic fluid of the earthworm *Aporrectodea molleri*, as well as those not treated (control) were used to extract lipid metabolites from the leaves after 60 days of culture.

### 3.1. Effect of isolates on plant-extracted metabolites

**Figure 1.**
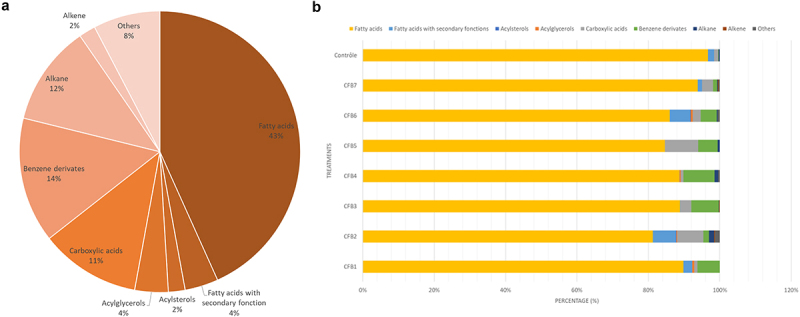
(a) Percentage (%) of metabolites identified by GC-MS that fall into each of the following 9 chemical categories. (b) Percentage (%) of different chemical classes in leaves of plants treated with coelomic fluid isolates (CFB1, …, CFB7) and untreated plants (control).

In order to investigate how plant inoculation with different CFB isolates affects their metabolic profile, we performed a metabolic analysis on leaf extract. This analysis identified a total of 104 distinct metabolites. We further categorized 104 metabolites into nine groups based on their molecular structure, as illustrated in Figure 1a.

Fatty acids were the most prevalent class of metabolites (45, or 43%), followed by benzene derivatives (15, or 14%), alkanes (12, or 11%), carboxylic acid and its derivatives (12, or 11%), fatty acids with secondary functions (4, or 4%) acylglycerols (4, or 4%) acylsterols (2, or 2%), alkenes (2, or 2%), and others (8, or 8%). We found 25 metabolites in untreated plants (control), compared to 52, 35, 32, and 31 in the leaves of plants treated with CFB2, CFB7, CFB4, and CFB6, respectively. In addition, 20 metabolites were identified in CFB1, 3 metabolites in CFB5, and 16 metabolites in CFB3 (Figure 1b).

Figure 1b shows that each treatment has a very high percentage of fatty acids across all 9 categories. Carboxylic acids were more prevalent in the CFB2 and CFB5 treatments when compared at the categorization level for each treatment. In general, only the leaves of plants treated with CFB2 and CFB6 had acylsterol-like substances, however, at deficient concentrations. Acylglycerols were only found in CFB1, CFB2, CFB4, and CFB6 treatments. The concentration of benzene derivatives was much greater in the CFB1, CFB2, CFB3, CFB4, CFB5, and CFB6 treatments. Acylglycerols, acylsterols, alkenes, and other metabolites were absent from the control in the form of metabolites.

**Figure 2.**
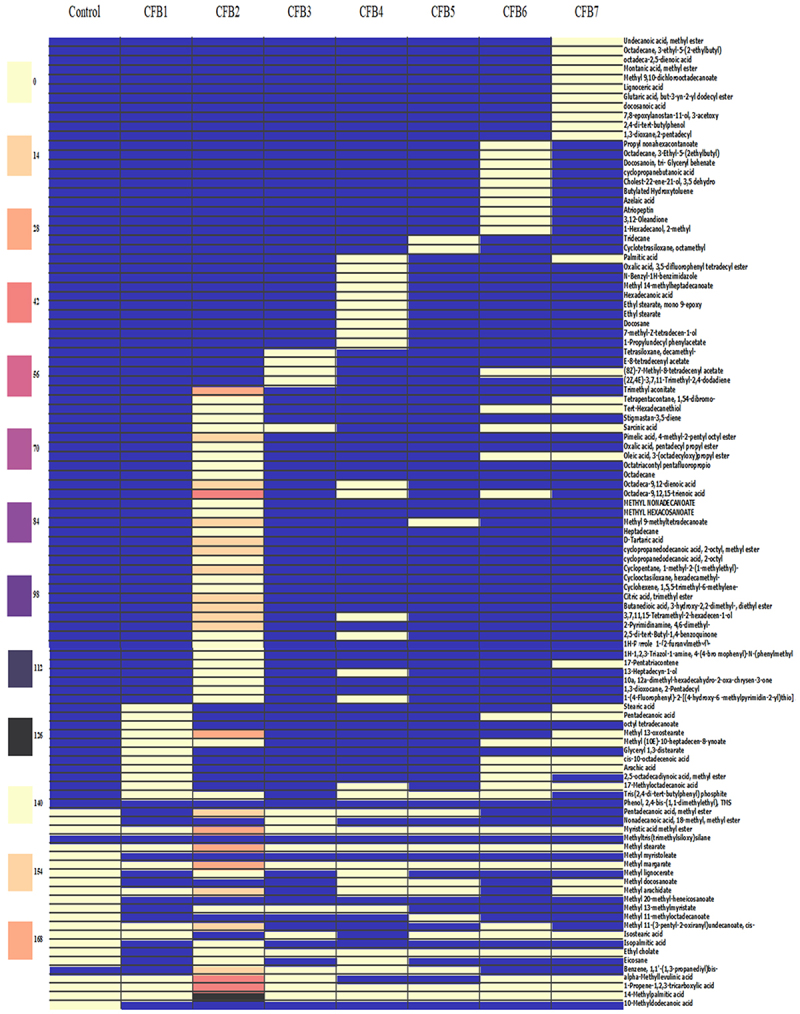
Metabolic profile obtained by GC-MS analysis for different bacterial treatments (CFB1, …, CFB7) and control treatment (control).

The heatmap analysis was conducted following the normalization of the data based on the metabolite’s abundance. The distribution of all metabolites throughout all treatments subjected to stress is depicted in Figure 2. According to a differential analysis, there were significant differences (*p* ≤ .05) in the chemicals between the various treatments. When plants were inoculated with CFB isolates, as opposed to the control, the variety of metabolites in the leaves rose, according to the metabolomic profile. It has been noted that a number of metabolites were absent from the control but were present in the leaf extracts that have been treated with CFB bacteria. Thus, the intensity of metabolites was significantly higher, especially in CFB2 treatment. At the level of CFB2 treatment, the following metabolites: methyl stearate, 14-methyl palmitic acid, alpha-methyllevulinic acid, octadeca-9,12,15-trienoic acid, 13-methyl oxosterarate, myristic acid methyl ester, methyl margarate, linoleic acid (octadeca-9,12-dienoic acid), methyl arachidate, methyl 9-methyltetradecanoate, pentadecanoic acid, methyl ester, were found at significantly very high concentrations, compared to all treatments. The figure indicated an absence of several benzoic compounds and alkanes with an absence of two classes of acylglycerols and acylsterols at the control level.

### 3.2. Effect of isolates on plant lipid composition

**Figure 3.**
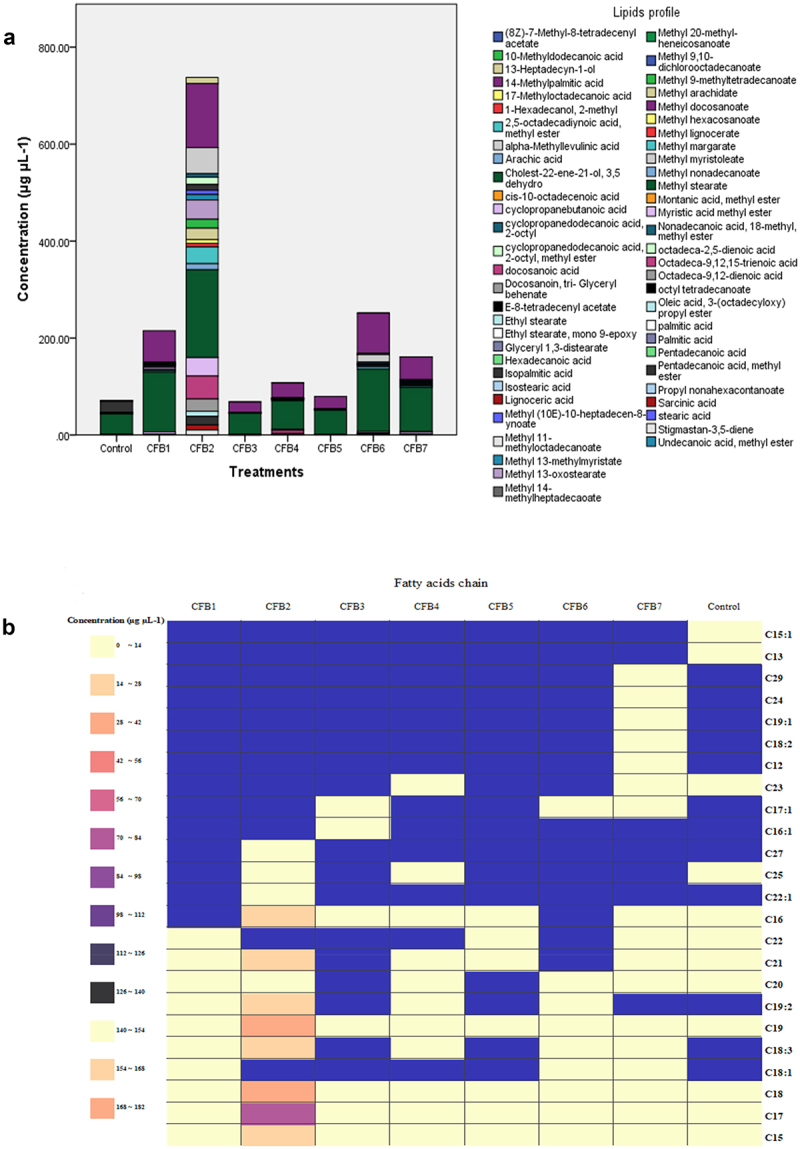
(a) The concentration of the different lipid compounds detected by GC-MS in each treatment (coelomic fluid bacterial treatments (CFB) and control treatment). (b) Fatty acid profile of the different coelomic fluid bacteria treatments (CFB) according to the length of their chain of carbon atoms.

To determine the impact of bacterial isolates on plant lipidomic, we simply compared the lipid profile of the leaves, as shown in Figure 3a. We noticed that there was a discernible change in composition between the CFB2 treatment and other treatments, as well as between the same treatment and the control. The CFB2 bacteria led to the appearance and the synthesis increase of a number of lipids. We found Methyl stearate and methyl palmitic acid in very high amounts in all treatments. Nonetheless, bacterial treatments, particularly CFB2 treatment, still had the highest concentration of these compounds. Based on lipid content and concentration, control was the treatment with the least variety.

The concentration of common compounds differed between control and bacterial treatments. The concentration of these metabolites was higher in bacterial treatments compared to control. The concentration of 14-methyl palmitic acid, methyl margarate, methyl stearate, and the methyl ester of myristic acid was 0.40, 1.17, 41.64, and 1.13 μg μL^−1^ while the concentration of the same compounds was 132; 34.56; 181.3 and 38.06 μg μL^−1^, respectively, in CFB2 treatment (Table 1).

**Table 1.** The common compounds between the 8 different treatments (the 7 coelomic fluid bacterial treatments (CFB) and control).

Concentration of common fatty acids (µg µL^−1^)		Treatments							
		Control	CFB1	CFB2	CFB3	CFB4	CFB5	CFB6	CFB7
14-methyl palmitic acid	0,3976	64,4771	132	21,5899	30,4623	25,6734	82,7914	46,4929	
Methyl margarate	1,1742	3,3514	34,56	1,1644	1,6621	1,3184	4,7673	2,8519	
Methyl stearate	41,6360	123,2254	181,3	42,5666	58,5766	49,2049	128,5337	90,9774	
Methyl ester of myristic acid	1,1267	4,2902	38,06	1,4025	2,2635	1,3927	3,5157	3,9364	

The CFB2 treatment increased the expression of these compounds in the plant by at least 93% (for example, methyl margarate).

**Figure 4.**
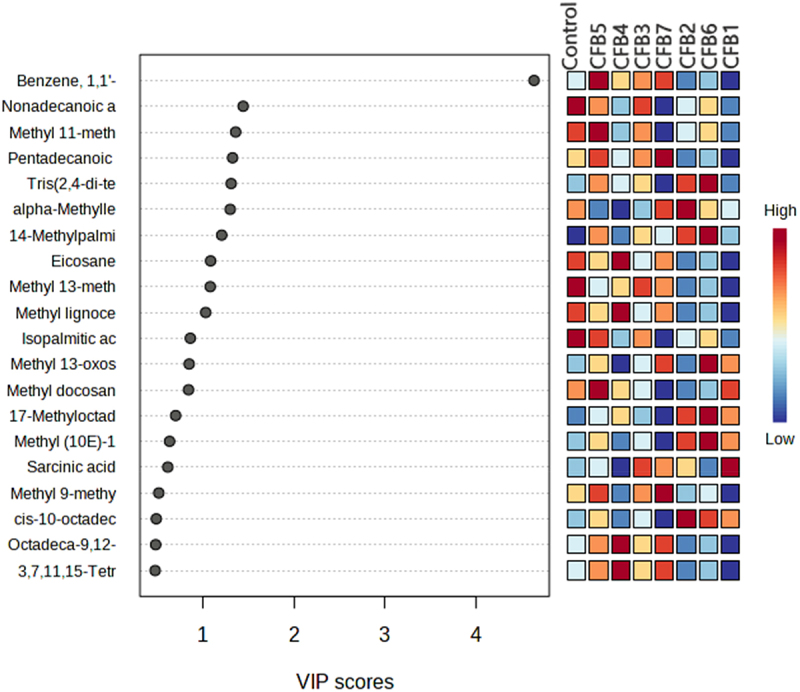
The top 20 most discriminating metabolites from the control, were ranked by variable importance in projection (VIP) scores. VIP scores > 1.0 were considered significant.

The relative contents of saturated fatty acids and unsaturated fatty acids with a long chain of carbon atoms (between 14 and 24 carbons), showed a significant accumulation in plants treated with CFB2, especially in methyl ester form (Figure 3b). In more detail, isopalmitic acid (C16:0), isostearic acid (C18:0), oleic acid (16-octadecenoic acid C18:1) and linolenic acid (9-octadecen-12-ynoic C18:3) significantly increased (*p* < .05) under CFB2 treatment. Additionally, the same treatment was observed to raise the concentration of methyl esterified forms of fatty acids, including myristic acid (methyl ester of myristic acid C15:0), palmitic acid (14-methyl palmitic acid C17:0), stearic acid (methyl stearate C19), arachidic acid (methyl arachidate C21:0) and linolenic acid (methyl ester of acid 10, 13-octadecadienoic C19:2). Based on “variable influence on the projection” (VIP) scores > 1, the top 20 metabolites that distinguished between treatments and controls were further determined (Figure 4). As the VIP value has a mean of 1, VIP values greater than 1 were typically regarded as important. A high score correlated with a great capacity for discrimination and served as a criterion selecting for biomarkers. After CFB4 and CFB3, Benzene, 1,1’-(1,3-propanediyl) bis- exhibited a greater VIP value and was significant for CFB5 and CFB2.

**Figure 5.**
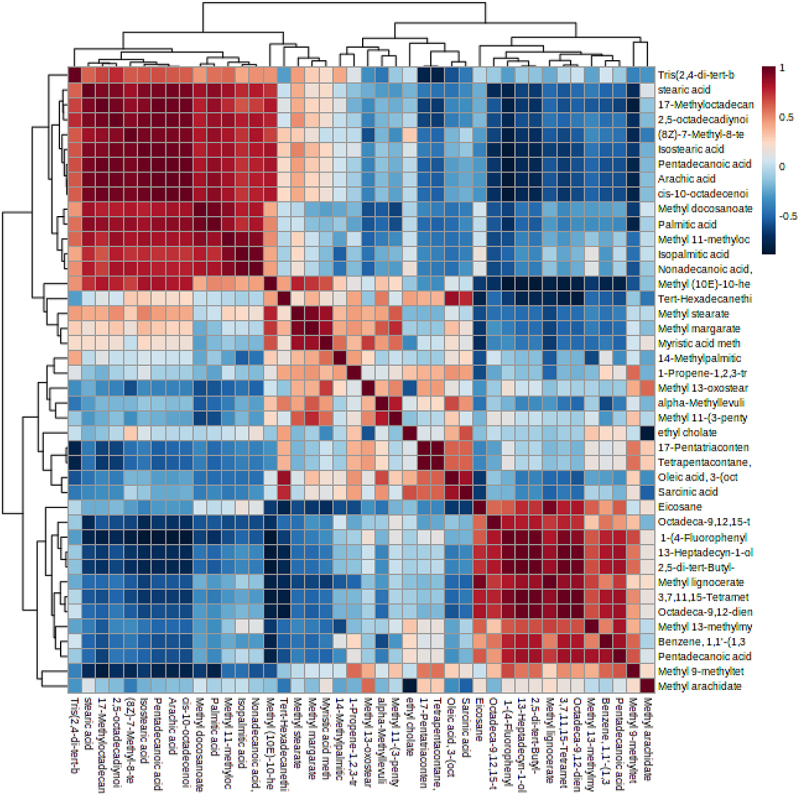
Heatmap analysis combined with hierarchical cluster analysis of the metabolomics in maize leaves under abiotic stress conditions. The correlation between different metabolites is shown by the intensity of the color in the heatmap. The heatmap was generated using Metaboanalyst online software.

Based on the correlation between the significant metabolites in various treatments (Figure 5), we could observe that two clusters of metabolites had a substantial positive connection between the metabolites of the same cluster. However, the correlation between the metabolites from different clusters was noticeably negative. The first cluster was primarily made up of fatty acids with long carbon chains. Along with the second cluster, which consisted mainly of short-chain carbon fatty acids, we also noticed a negative correlation between long and short fatty acid chains. Surprisingly, the intensity variation in the correlation between the benzoic compounds (heptadecyn-1-ol and di-tert-butyl) and other metabolites was the same.

### 3.3. Multivariate data analysis

**Figure 6.**
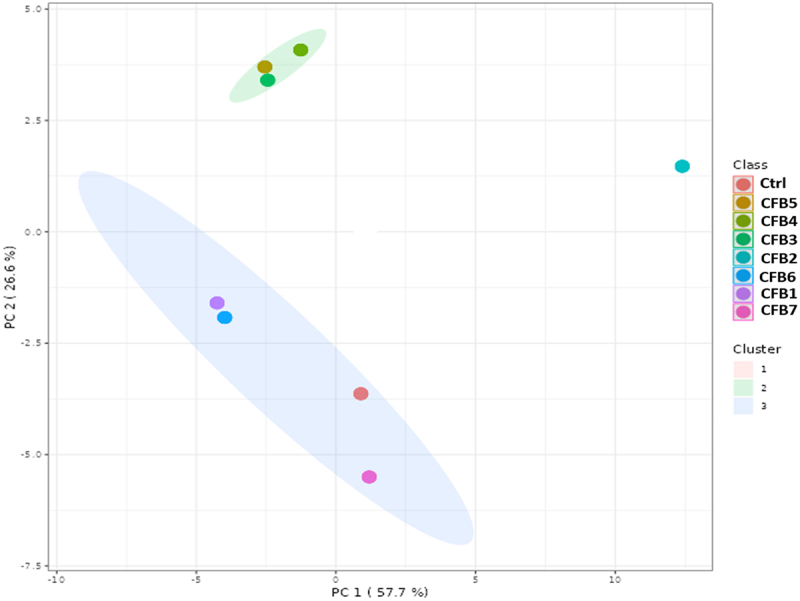
Principal component analysis (PCA) of different treatments (coelomic fluid treatments: CFB and control: Ctrl). The PCA analysis was constructed using K-means.

To minimize the dimensionality of the data and depict sample grouping, the GC-MS data collected from all treatments were subjected to an unsupervised multivariate data processing approach called PCA. The PCA analysis using K-means in Figure 6 illustrates how the metabolomic profiles of various treatments vary. Two principal components (PC1 and PC2) were used to generate the PCA model, and they had predictability values of 26.6 and 57.7%, respectively. According to the PCA results for the eight samples, 3 clusters were clearly separated from one another. There was no discernible difference between the CFB6 and CFB1 treatments and the CFB5, CFB4, and CFB3 treatments. The control and CFB7 were positioned close to one another in the same cluster. The only treatment that was placed in a separate cluster (cluster 1) was CFB2, showing its completely distinct impact on the plant lipidome.

### 3.4. Correlation between plant metabolites and growth parameters

**Figure 7.**
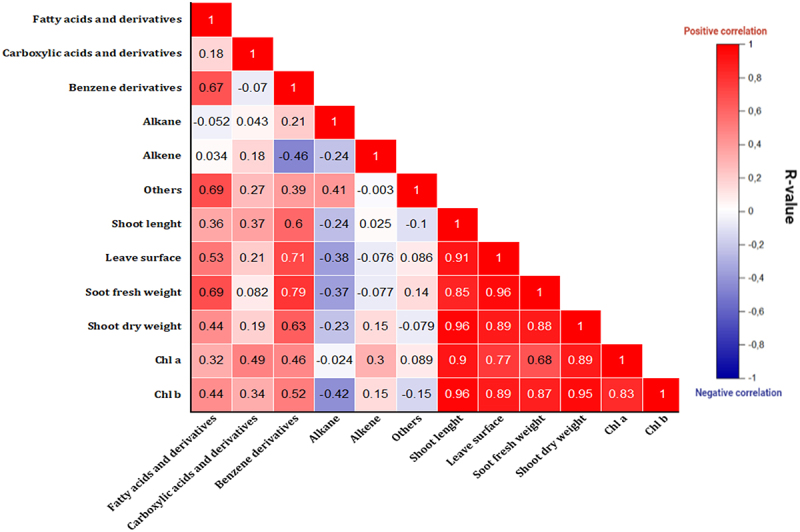
Correlation between the different classes of metabolites (fatty acids and derivatives, carboxylic acids and derivatives, benzene derivatives, alkanes, alkenes, and others) and plant growth parameters (from our previous study) such as length of the aerial part, leaf surface, biomass (fresh and dry weight of the aerial part) and chlorophyll content (a and b).

Finally, in order to understand the correlation between plant metabolites and plant growth parameters, we have conducted a heatmap analysis. Figure 7 illustrates the connection between the structure of the extracted metabolite concentration data and the plant growth parameters (from our previous study) using a heatmap representation. Our earlier investigation, carried out simultaneously with the current study, evaluated the impact of CFB on the plant growth under the same abiotic stress conditions. Compared to maize cultivated in uninoculated soil, the effect of CFB on boosting plant growth under abiotic stress was substantially exceptional. CFB increased root and shoot length. Furthermore, the presence of isolates in the soil resulted in a significant increase in plant fresh and dry weights. The heatmap generated during the current investigation revealed that metabolites from fatty acids and derivatives, carboxylic acids and derivatives, benzene derivatives, and alkanes were directly correlated with plant growth parameters such as the length of the aerial part, the surface of the leaves, and the biomass (fresh and dry weight of the aerial part). However, they had a negative correlation with alkenes and other metabolites.

## 4. Discussion

It has already been revealed that earthworms stimulate plant growth and yield under abiotic stress.^32,33^ However, the contribution of its associated bacteria to this process has yet to be fully understood or appreciated.^34^ Recently, the earthworm’ coelomic fluid (CF) has attracted interest because of its potential to increase seed germination and growth parameters.^16,17^ In addition, and for the first time, our previous study showed a significant improvement in plant growth in the presence of *Bacillus* (CFB6: *Bacillus paramyides* and CFB7: *Bacillus thuringiensis*), *Pseudomonas* (CFB4: *Pseudomonas aeruginosa*), of *Buttiaxella (*CFB1: *Buttiauxella gaviniae*), *Pantoea* (CFB2: *Pantoea vagans*), *Aeromonas* (CFB3: *Aeromonas hydrophila*), and *Staphylococcus* (CFB5: *Staphylococcus epidermidis*).^15,35^ In addition to stimulating plant growth, CFB isolates increased plant moisture content compared to control. Furthermore, decreased plant growth and yellowing of leaves caused by reduced chlorophyll production, as symptoms of alkalinity and nutrient deficiency conditions, were observed in soil not inoculated with bacteria.

We conducted the present study to evaluate, for the first time, the effect of seven bacterial strains isolated from the CF of the earthworm *Aporrectodea molleri* on plant metabolism under conditions of abiotic stress (nitrogen, phosphate, and potassium deficiency combined with soil alkalinity). The results of the metabolomic analysis revealed the presence of metabolites that are expressed in bacterial treatments and absent in control and others that are common but are found at very high concentrations compared to control. This implies that bacteria isolated from the coelomic fluid could improve plant growth and stress tolerances by intervening in the activation of metabolic pathways.

The heatmap analysis (Figure 7) between the leaf metabolites of plants treated with CFB and their growth parameters showed that the length of the aerial part, the surface of the leaves, and the biomass (fresh and dry weight of the aerial part) had a positive correlation with specific metabolites belonging to fatty acids and derivatives, carboxylic acids and derivatives, benzene derivatives, and alkanes. The possible explanation for this finding is that positively correlated metabolites are positive signals regulating plant growth and resilience to stress (NPK nutrient deficiency and soil alkalinity). The fact that the metabolites common between all treatments are present in high concentrations in bacterial treatments compared to the control treatment implies that the bacteria stimulated the overexpression of these metabolites. Isolates CFB2 (*Pantoea vagans*), CFB4 (*Pseudomonas aeruginosa*), CFB6 (*Bacillus paramyides)*, and CFB7 (*Bacillus thuringiensis*) resulted in significantly significant production of various metabolites belonging to the different chemical categories.

Natural benzene byproducts may be present in the composition of lignine, suberine, pigments (flavonoids, anthocyanins), and other phenolic compounds.^36^ The amount of these metabolic products observed in bacterial treatments could indicate that their production pathway is being upregulated.

The sterols and glycerol found only in bacterial treatments (CFB1, CFB2, CFB4, and CFB6) (Figures 1b and 2) control the cellular uptake of nutrients, which plays a significant role in plants’ growth and tolerance to biotic stress.^37^

Three hydrocarbon compounds that are typically found in plants in response to stressful situations include heptadecane, octadecane, and azelaic acid.^38^ These metabolic processes are concentrated at high levels in plants treated with coelomic isolators, which likely increases their tolerance.

Other mechanisms, such as adjustments to the membrane’s fluidity and adjustments to the membrane’s lipid composition which are greatly mediated by desaturases, are also included in the responses of the tolerance system.^39^ The primary components of cellular membranes are lipids, such as fatty acids. The involvement of free fatty acids in cellular signaling has been linked to various responses to stress, plant growth, and plant development. Moreover, the concentrations of unsaturated fatty acids play a crucial role in the interactions between plants and microorganisms.^39–42^

This study’s lipidomic analysis by GC-MS revealed an increase in the concentration of unsaturated fatty acids (Figure 3b), particularly linolenic acid (C18:3). In fact, it has been suggested that galactolipids with high concentrations of long-chain, polyunsaturated fatty acids, such as linolénic acid, are responsible for the fluidity of chloroplast membranes and contribute to photosynthetic activity. According to Phamthi (1984), changes in the lipid composition of plant leaves in response to biotic stress were related to changes in photosynthetic activity, which explains why the concentration of chlorophyll in plants treated with CFB was not affected by stress as it was in the case with the control. Thus, it is obvious to suggest that the CFB treatment had a positive impact on the C18:3 related to photosynthetic activity. The findings of this study also showed an increase in the use of stearic acid, palmitic acid, oléic acid, myristoleic acid, and arachidonic acid in bacterial treatments. Indeed, it is known that the palmitic and oleic acids (16:0 and 18:0) can aid in the growth and development of plants grown in stressful environments.^43^

The whole set of extracted and processed metabolites from the KEGG database shows that the isolates triggered the production of fatty acids. The production of fatty acids in plants involves a number of condensation reactions involving the enzymes acetyl-CoA carboxylase (ACC) and acetyl-CoA synthase. These reactions result in the synthesis of C16:0, which can be extended to C18:0 and become desaturated.

These fatty acids, beyond their role in membrane function, play a crucial role in plant survival. They can be stored in cellular membranes, reducing water flow, and providing a protective barrier against microbial stress, thereby enhancing the plant’s resilience.

As a result, the increase in C16:0 and C18:0 seen in this study may indicate the synthesis of membrane lipids, which are a complex mixture of long-chain fatty acids (C20 to C40) and their derivatives.^27,44,45^

As plants are exposed to various forms of stress, lipid regulation, synthesis, degradation, relocalization, or restructuration they frequently undergo remarkable changes.^42^ In reality, there is a lack of knowledge on how alkaline soil affects plant growth and metabolism. Nutrient deprivation is a well-studied biological stress.^42^ Many nutrients influence the homeostasis of lipids, but phosphorus is the most significant and most researched after carbon (P). The degradation of phospholipids during P deficiency allows plant cells to use the released P.^42^ The fact that the CFB treatment of the plants did not result in a decrease in the content and concentration of lipids proves that the plants were not negatively impacted by abiotic stress (in contrast to the control), demonstrating the rigidity of the protective mechanisms.

According to earlier research, the overexpression of certain metabolic enzymes involved in the tricarboxylic acid (TCA) cycle, as well as the pathways for glyoxylate and dicarboxylate metabolism in plants may alter certain biological processes, including the transmission of hormonal signals and the plant’s tolerance to abiotic stress.^46^ The metabolism of glyoxylate and dicarboxylate plays a crucial role in resolving the metabolic issues that plants face and in the transportation of energy to increase the plant’s tolerance to stress.^46^ According to the present study, bacterial treatments had higher concentrations of the substances related to glyoxylate and dicarboxylate metabolism.

The effects of abiotic stress on plants include complicated responses, including various signaling events, physiological adjustments, and the activation of defense mechanisms that collectively alter the biosynthesis, transport, and storage of several primary and secondary metabolites.^47^ Several forms of experimental evidence have shown that stress-related biological factors can affect the levels of the metabolites involved in the cycle of endogenous tricarboxylic acid (TCA) in plants.^47^ The metabolic level of TCA is closely correlated with root development, phosphorus deficiency, and abiotic stress; however, the molecular functions of TCA genes are yet unknown.^47^ During aluminum stress, there was a significant increase in the levels of TCA metabolites in *Zea mays* (ma) root exudates. Additionally, it has been reported that plants growing in alkaline soils secrete compounds from their root’s TCA pathway, which enables them to absorb vital nutrients like phosphorus and iron by lowering the pH of the rhizosphere.^47–49^ The activity of the pathways involved in increasing tolerability, which was higher in plants treated with CFB than in plants under control, suggests that the treated plants’ aerial parts suffered less severe damage than the untreated ones.

## 5. Conclusion

The greenhouse culture of maize plants treated with plant growth-promoting isolates and cultivated in alkaline soil lacking N, P, and K have shown that coelomic fluid bacteria (CFB) greatly increase plant resistance to abiotic stress. We might conclude that earthworm-associated bacteria have a strong potential for enhancing plant metabolism to withstand adverse environmental conditions. The rigidity of the defensive systems of the treated plants is shown by the fact that the content and the concentration of lipids did not change, showing that the plants were not adversely affected by abiotic stress. The increased concentration of long-chain polyunsaturated fatty acids, which are necessary for the fluidity of chloroplast membranes, allowed plants to maintain their photosynthetic activity.

In addition, CFB treatment positively impacted stearic acid, palmitic acid, oleic acid, myristoleic acid, and arachidonic acid concentrations involved in the growth and development of plants grown in stressful environments. Bacterial treatments can also increase maize tolerability by stimulating the metabolism of the glyoxylate and dicarboxylate pathways. However, further research could explore the specific mechanisms by which the earthworm-associated bacteria enhance plant production of beneficial fatty acids. Additionally, investigating the potential application of CFB treatment in crop cultivation under different abiotic stress conditions could provide valuable insights for increasing crop resilience.

## Data Availability

The data used in the present work are available and presented within the article.

## Statement

“We confirm that experimental research and field studies on plants (either cultivated or wild), including the collection of plant material, complied with relevant institutional, national, and international guidelines and legislation.”

“No approvals were required for the study, which complied with all relevant regulations.”
